# Vinculum of Cardiovascular Disease and Inflammatory Bowel Disease: A Narrative Review

**DOI:** 10.7759/cureus.26144

**Published:** 2022-06-21

**Authors:** Ashujot Kaur Dang, Daniel A Gonzalez, Rajeswar Kumar, Saba Asif, Anoushka Bali, Krishna Kishore Anne, Nithin Kumar Konanur Srinivasa

**Affiliations:** 1 Research, Government Medical College, Patiala, Patiala, IND; 2 General Physician, Universidad Catolica Santiago de Guayaquil, Guayaquil, ECU; 3 Medicine, Rajah Muthiah Medical College, Chidambaram, IND; 4 Internal Medicine, Apollo Hospitals, Hyderabad, IND; 5 Research, Acharya Shri Chander College of Medical Sciences & Hospital, Jammu, IND; 6 Internal Medicine, National Pirogov Memorial Medical University, Vinnytsya, UKR; 7 Research, Bangalore Medical College and Research Institute, Bangalore, IND

**Keywords:** heart failure, peripheral vascular disease, ischaemic heart disease, ischemic cerebrovascular disease, immune-mediated injury, crohns, ulcerative colitis, venous and arterial thrombosis, cardio vascular disease, inflammatory bowel disease

## Abstract

Inflammatory bowel disease (IBD), comprising of ulcerative colitis (UC) and Crohn's disease (CrD), is a chronic relapsing-remitting inflammation of the bowel with extraintestinal involvement. Numerous studies published in the last decade have underlined the dangerous cardiovascular disease (CVD) outcomes of IBD, such as ischemic heart disease, heart failure, and stroke, and the need for better therapeutic and prognostic strategies. This article elucidated the pathological web of mechanisms that link IBD with CVD, such as immune dysregulation, endothelial dysfunction, arterial stiffness, and dysbiosis, with a comprehensive review of clinical studies standing for and against the notion in pediatric and adult populations. The current treatment and prevention aim at disease remission and dietary strategies shown to reduce the CVD risk. Exploration of other supplemental preventive and treatment methods, especially during active flares of disease, to reduce the risk of arterial thromboembolic disease (ATED) is the need of the hour.

## Introduction and background

The term inflammatory bowel disease (IBD) refers to a chronic relapsing systemic inflammatory condition that consists of two separate entities: ulcerative colitis (UC) and Crohn's disease (CrD), which are primarily present with gastrointestinal symptoms but may have extra gastrointestinal manifestations as well. Specific symptoms are common to UC and CrD, such as fever, weight loss, abdominal pain, and bloody or non-bloody diarrhea. However, UC causes superficial ulcerations on the mucosa of the colon; on the other hand, CrD presents with transmural inflammation and granulomas along with fistulas and strictures. CrD can involve any part of the GI tract, but it is common in the ileocecal region. Patients with IBD can also present with extraintestinal symptoms like arthritis, uveitis, and erythema nodosum. In pediatric patients with IBD, there is a possibility of growth impairment, delayed puberty, and failure to thrive. In pediatric patients with IBD, there is a possibility of growth impairment, delayed puberty, and failure to thrive [1,2].

The epidemiological burden of the disease is reaching new heights, with an incidence rate of 1.4 per 100,000 in Asia [3], and nearly 2.2 million Europeans [4] and 7.7 million Americans afflicted by it [5]. Several chronic inflammatory diseases have previously been associated with an elevated risk of cardiovascular disease (CVD), including rheumatoid arthritis (RA) and systemic lupus erythematosus (SLE) [6-8]. However, the extent of CVD risk in IBD patients has been controversial. Interestingly, even though IBD patients have a lower incidence of classical risk factors such as high body mass index (BMI) or hyperlipidemia compared to the general population, the overall CVD incidence is at elevated levels in IBD patients despite the possibility of early detection and adequate prophylaxis for CVD as they enter the healthcare system at an earlier age. Given that IBD patients usually lack the traditional risk factors associated with cardiovascular disease, it is reasonable to speculate that certain other pathological factors may contribute to rising CVD risk [9,10]. Recent literature has shown that IBD patients are prone to subclinical atherosclerosis, resulting in arterial stiffness due to chronic inflammation leading to endothelial dysfunction, promoting platelet aggregation and plaque formation [11,12].

Moreover, the literature has moved beyond atherosclerosis being a passive process of cholesterol build-up in arteries to an inflammatory and immune-mediated process of plaque build-up and destabilization [11,12]. Furthermore, the gut microbiota itself may influence the development of atherosclerosis in IBD patients [9]. Researchers in recent years have explored the possibility of ischemic heart disease (IHD), heart failure (HF), and other CVDs in patients with IBD. Effective management of IBD may reduce CVD risk, but the research to support this recommendation is limited. Certain anti-inflammatory drugs like steroids could also increase CVD risk [8]. Despite the potential link between atherosclerosis, thrombosis, and increased CVD risk in IBD patients, the pathological pathways are poorly understood. The pathological risk factors have not yet been well defined clinically. This article aims to present the current concepts and studies to the best of our knowledge and highlight the knowledge gap in the contemporary literature that explores CVD risk in IBD patients, both in adult and pediatric populations.

## Review

Mechanisms that interconnect IBD and CVD

Inflammation and Immune Dysregulation

**Figure 1:**
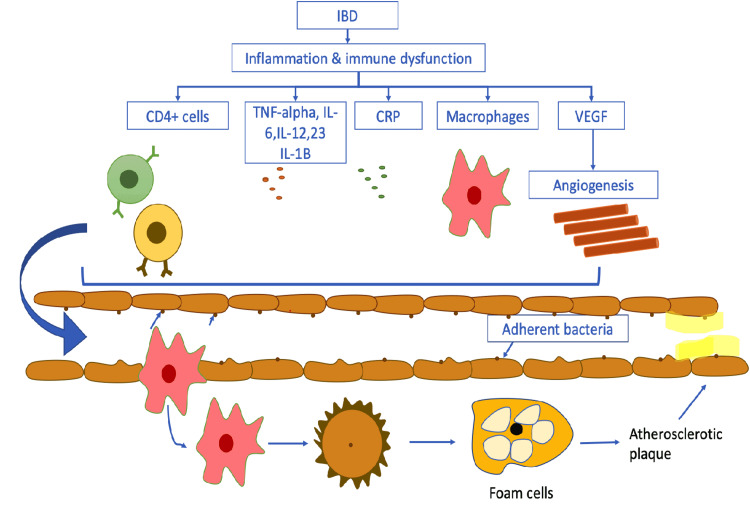
Pathogenesis of inflammation and immune dysfunction leads to atherosclerotic plaque formation. Image Credits-Ashujot Kaur Dang. IBD: inflammatory bowel disease; CD: cluster of differentiation; TNF: tumor necrosis factor; IL: interleukin; CRP: C-reactive protein; VEGF: vascular endothelial growth factor.

An intriguing concept in the development of atherosclerosis in chronic inflammatory diseases is the role of inflammatory cytokines and inflammatory cells in catalyzing and enduring inflammation in the microenvironment of the atherosclerotic plaque [13]. The pathogenesis of the association between IBD and atherosclerotic cardiovascular disease (ASCVD) has not been entirely determined in detail. However, the latest advancements in science show that low-grade chronic inflammation is implicated in all phases of atherosclerosis, from the initiation phase to the catenation and future complications of atherogenesis [14]. There are two ways that atherosclerosis can develop: a direct mechanism that occurs in situ in the vessel wall and an indirect one that occurs due to the release of cytokines from non-vascular sites, accelerating the development of atherosclerosis (Figure 1) [15].

Intestinal inflammatory diseases often cause mucous layer disruption and tight junctions malfunctioning. This malfunctioning results in increased permeability of cells and strong adhesion by bacteria to epithelial cells, increasing the bacterial load. IBD is characterized by the production of pro-inflammatory cytokines such as tumor necrosis factor (TNF)-α, interleukin (IL)-1β, interleukin-6, interleukin-12, interleukin-23, and chemokines by the innate immune cells (Figure 1). Inflammatory reactions are further escalated when these chemokines activate clusters of differentiation (CD)4^+^T cells, leading to an increase in cytokines and the recruitment of other leukocytes further. This nexus of events leads to inflammation and tissue damage [16,17]. Inflammatory cytokines produced by IBD are also thought to cause endothelium-dependent dilatation of blood vessel walls, increasing the risk of atherosclerosis (Figure 1) [18]. Elevated levels of C-reactive protein (CRP) and IL-6 are independent risk factors for CVD as they promote the formation of new plaques and rupture of existing plaques, which is also why excessive levels of CRP are predictive of CVD risk [19,20]. Angiogenesis and inflammation are influenced by the release of the vascular endothelial growth factor (VEGF) in people with existing IBD, making atherosclerosis worse [21]. The following flow chart illustrates the pathogenesis in a simple way (Figure 1).

Endothelial Dysfunction

Chronic ongoing inflammatory conditions like IBD alter endothelial cells' physiological functions, leading to endothelial dysfunction (ED). ED is notorious for vasoconstriction, which occurs due to increased smooth muscle tone, pro coagulation, angiogenesis, and increased leukocyte cellular adhesion, leading to leukocyte diapedesis. As a result, ED is one of the most significant independent risk factors for developing ASCVD [18]. Physiologically speaking, the average healthy endothelium, the innermost layer of blood vessels, controls arterial tone and structure by releasing substances that act as anticoagulants, antiplatelets, and fibrinolytic in action [22]. The endothelium releases nitric oxide (NO) and prostacyclin, which act as vasodilators and inhibit platelet aggregation, thus working as antiplatelet agents. It also releases bradykinin, which stimulates tissue plasminogen activator (tPA), a fibrinolytic agent. Conversely, bradykinin stimulates the release of NO and prostacyclin, further enhancing its antiplatelet effects [23]. However, this antiplatelet action becomes disrupted in ED when inflammation damages the endothelium, as a result, increases the permeability into the vessel wall, promotes platelet aggregation, and increases leukocyte adhesion and release of cytokines in huge numbers due to inflammation. The endothelial damage consequently releases all the intrinsic substances produced by the endothelia, which upsets the balance between vasoconstrictors and vasodilators, leading to the nexus of events that commence, catenate, and congregate plaque formation leading to ASCVD [23].

**Figure 2:**
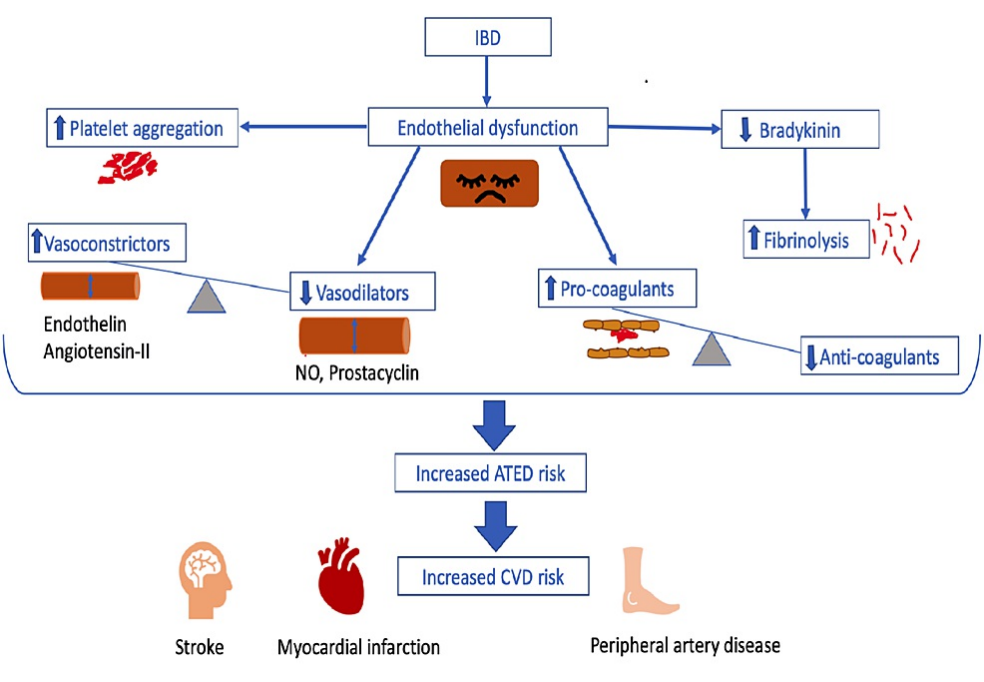
Pathogenesis of the endothelial dysfunction caused by the ongoing chronic inflammation leads to increased cardiovascular risk. Image Credits- Ashujot Kaur Dang. IBD: inflammatory bowel disease; ATED: arterial thromboembolic disease; CVD: cardiovascular disease; NO: nitric oxide.

Endothelial cells produce substances that constrict blood vessels in these circumstances, including endothelin, which is the most potent vasoconstrictor identified to date, and angiotensin II. A combination of endothelin and angiotensin II also contributes to plaque formation by promoting the proliferation of smooth muscle cells, thereby increasing the risk of atherosclerosis [23]. Moreover, NO's reduced production and release results in impaired vasodilation, an early indicator of atherosclerosis [24]. The decreased NO production that occurs in IBD could have been a result of increased arginase activity that competes with the nitric oxide system (NOS) and reduces the NOS messenger ribonucleic acid (mRNA) expression [25]. On the other hand, angiogenesis is climacteric for nurturing the ongoing chronic inflammation in the gastrointestinal tract. The influx of angiogenic factors and pathological angiogenesis in inflammatory tissues is regulated by several inflammatory cells, including macrophages, mast cells, lymphocytes, and fibroblasts (Figure 2) [25,26]. The lack of oxygen in the inflamed area also invigorates angiogenesis by increasing the release of VEGF, fibroblast growth factor, and TNF-α [26]. Figure 2 below embellishes endothelial dysfunction's pathogenesis, leading to an increased risk of cardiovascular diseases in the IBD population.

Arterial Stiffness

**Figure 3:**
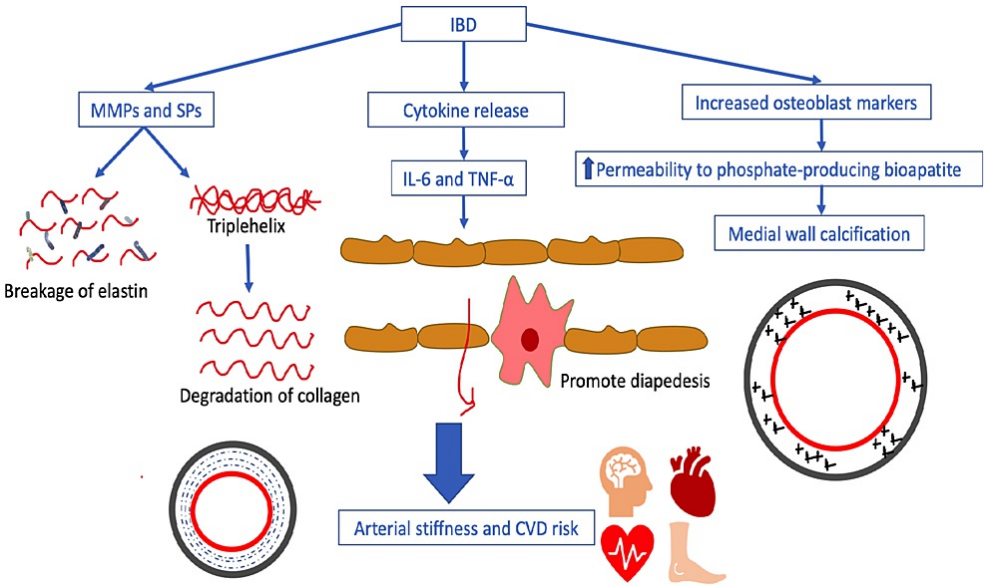
Pathogenesis of chronic ongoing inflammation leads to arterial stiffness and thereby increased CVD risk. Credits- Ashujot Kaur Dang. IBD: inflammatory bowel disease; MMPs: matrix metalloproteinases; SPs: serine proteinases; IL: interleukin; TNF: tumor necrosis factor; CVD: cardiovascular disease.

In chronic inflammatory diseases, the relapsing and remitting episodes of inflammation with acute flare-ups can damage the walls of the arteries. Although several risk factors are notorious for causing arterial stiffness (AS), systemic inflammation is one such risk factor [27]. It is important to note that AS may occur independently of atherosclerosis in IBD patients even without conventional risk factors for CVD [28]. Additionally, it is linked to the inflammatory disease's duration and severity [29]. As mentioned above, ED is associated with the proliferation of vascular smooth muscle cells and chronic inflammation, which predisposes to vascular remodeling and increased collagen synthesis, invigorating AS (Figure 3) [28].

In IBD, the cytokines released in huge numbers promote diapedesis into the blood vessel wall, predominantly mediated by IL-6 and TNF-α and change the smooth muscle cell phenotypes by releasing matrix metalloproteinases (MMP) and serine proteinases (SP) (Figure 3). This activation of MMPs and SPs leads to increased breakage of elastin macromolecules. In addition to elastin degradation, MMPs also disintegrates collagen stands, rendering the collagen uncoiled and disorganized, making it inflexible [30].

In the microenvironment of inflammation, osteoblast markers on vascular smooth muscle cells in the arterial walls make them permeable to taking up phosphate-producing bioapatite. This bioapatite ensues medial wall calcification reducing the vessel elasticity and leading to AS (Figure 3) [31]. CRP, a typical active phase reactant, has an active incrimination in invigorating endotheliitis and endothelial dysfunction. Inflammation of the vessels providing oxygen to the vessels themselves (called vasa vasorum) can ensue in the vessel ischemia catenating further the remodeling of the connective tissue matrix leading to AS [27]. The CD40-CD40‐ligand system between the helper T cells and endothelial cells is also climacteric in mediating leukocyte-endothelial interactions in the inflamed intestine leading to immune-mediated damage and dysfunction [32]. Figure 3 elucidates the AS and its role in the pathogenesis of CVD.

Microbiota Dysbiosis

The gut microbiota is a fundamental component of the human body. Its altered composition, also known as dysbiosis, has recently been implicated in the underlying mechanisms of many diseases, including chronic inflammatory diseases like IBD [33]. The disintegration of intestinal microbiota in genetically susceptible individuals induces an abnormal immune response that violates the intestinal homeostasis leading to escalated inflammation and severity of IBD. Also, the increased inflammation conversely damages the mucosal layer of the intestine, directly affecting microbiota composition, leading to further dysbiosis [34].

The dysbiosis that occurs due to IBD is also implicated in atherosclerosis and arterial stiffness development. The culprit behind it is found to be the metabolites secreted by the altered gut bacterial composition. There are significant alterations in the gut microbiota in terms of numbers and types of bacteria. There is a decreased number of commensal bacteria, such as *Bacteroides*, *Prevotella,* and *Fecalibacterium,* whereas *Enterobacteriaceae* including *Escherichia coli* are found to be increased in CrD patients [33]. The latter gut bacteria are notorious for secreting indole and phenyl derivatives that fuel plaque formation and further exacerbate atherogenesis, increasing CVD risk [35]. Furthermore, indole and its other derivatives have been found to interfere with the serotonin signaling pathways, affecting arterial blood pressure via central and peripheral mechanisms. The phenyl derivatives of hippuric acid have been associated with adverse cardiovascular events and outcomes [35].

Another pathway that increases CVD risk like coronary artery disease (CAD) and IHD in IBD patients is the disjunction of intestinal cells' inter-epithelial tight junctions due to altered microbiota composition, thereby disrupting the barrier and leading to increased permeability of wanted and unwanted substances. This dysfunctional barrier permits the increased absorption of endotoxins and lipopolysaccharides (LPS) and increased bacterial load, leading to escalated chronic systemic inflammation, especially of the vascular endothelium, further expediting and catalyzing the process of atherosclerosis. Furthermore, LPS instills a robust innate immune response with the release of pro-inflammatory cytokines and chemokines that increase low-density lipoprotein (LDL) oxidation and macrophage activation, leading to the formation of foam cells, thereby increasing plaque formation in the arteries [36].

Clinical implications

It has already been established that IBD is a prothrombotic state giving rise to venous thromboembolic disease (VTED), and many treatments and precautions are already well understood and followed in IBD subjects. However, the implications of arterial thromboembolic diseases (ATED) and other cardiovascular events in IBD are still controversial [37].

There is substantial heterogeneity in the current data concerning the risk of arterial thromboembolic events in IBD patients, including cerebrovascular accidents (CVAs), IHD, and myocardial infarction (MI). Also, the relationship between IBD and heart failure (HF) is ambiguous [38].

Cerebral ATED and IBD

As explained above, IBD patients are at an increased risk of thrombi formation in both veins and arteries due to a hypercoagulable state owing to endothelial dysfunction and other pathophysiologies. Existing clinical research underlines the propensity to form thrombi or emboli in cerebral arteries leading to CVA and stroke to indicate the intensity of the risk of ATED in IBD adult and pediatric populations.

Bernstein et al. performed a case-control study published in 2008, sourcing data from the University of Manitoba database found that the risk of cerebral ATED is higher in CrD [39]. In comparison, another contrasting retrospective cohort study with 17,487 IBD patients and 69,948 controls highlighted the female predominance. Both CrD and UC patients under 40 years of age were at a twofold higher risk of cerebral ATED. However, men with IBD did not share these risks [40] (Table 1). Furthermore, another study by Andersohn et al. narrated that CrD patients below 50 years of age had a slightly higher risk of stroke (0.99 OR; 95%CI, 0.75-1.30) than older patients but showed no specific gender predominance [41]. A meta-analysis and systematic review that Singh and his colleagues conducted analyzed nine studies that compared the incidence of CVA and IHD in IBD patients compared with the standard non-IBD population. IBD was associated with a modest increase in CVA incidence, particularly in females and young age patients (40-50-year-olds) [42]. These studies were also supported by evidence from Kristensen et al. that confirmed the increased risk of CVA, especially during active flares and persistent IBD [43]. Howbeit, in contrast to the studies above, Sridhar et al. also analyzed and investigated CVA and other CVDs but did not find any increased risk due to IBD in their crossectional study; instead, they found a reversed relationship between these diseases and IBD [44] (Table 2). A study conducted by Barclay and his team on 154 pediatric newly diagnosed IBD patients, 56% of which were males and the rest females, and subgroup divisions as UC 30%, CrD 64%, IBD unclassified (IBD-U) 6%, studied the incidence and outcome of CVA over five years and also the relative proportion of stroke reported in the literature in patients with UC and CrD before and after January 2000. This study found that all patients had a risk factor for CVA, and four cases of CVA (2.6%) were recognized out of the lot. It also highlighted a significant increase in the relative proportion of strokes in CrD cases reported after January 2000 [45].

**Table 1:** Studies supporting the association between inflammatory bowel disease and cardiovascular disease. IBD: inflammatory bowel disease; ATED: arterial thromboembolic diseases; UK: United Kingdom; CrD: Crohn’s disease; IHD: ischemic heart disease; CVA: cerebrovascular accident; CVD: cardiovascular disease; VTED: venous thromboembolic disease; UC: ulcerative colitis; CAD: coronary heart disease; PCI: percutaneous coronary intervention; CV: cardiovascular; NIS: National Inpatient Database.

Author name and reference	Year of publishing	Design	Cases of IBD	Cases not with IBD	Population	Conclusion
Bernstein et al. [39]	2008	Case-control study	8060	80,849	University of Manitoba IBD database	Cardiac and cerebral ATED were higher in the IBD group
Ha et al. [40]	2009	Retrospective cohort study	17,487	69,948	Thomson Reuters MarketScan research database	Higher risk for mesenteric ischemia in the IBD group and higher risk of ATED in IBD females
Andersohn et al. [41]	2010	Case-control study	8054	161,078	UK general practice research database	Higher risk of stroke in younger (<50 years) CrD patients
Singh et al. [42]	2014	Systematic review and meta-analysis			Nine cohort and case-control studies	19% increased risk of IHD and also a higher risk of stroke, especially in women
Kristensen et al. [43]	2013	Retrospective cohort study	20,795	199,978	Danish national registers	Overall increased risk of CVD, including IHD, CVA, and cardiovascular death in IBD
Kristensen et al. [46]	2014	Prospective cohort study	23,681	5,412,966	Danish citizens	Patients with IBD had a 37% increased risk of hospitalization for HF
Fumery et al. [47]	2014	Meta-analysis			Thirty-three studies enrolling 207,814 IBD patients and 5,774,898 controls	Increased risk of VTED and IHD while no increased risk of ATED or CV mortality
Aggarwal et al. [48]	2014	Cohort study	131	524	Patients with IBD who were diagnosed with CAD by cardiac catheterization	CAD at an early age in the IBD group but post-PCI outcomes similar to the non-IBD group
Yarur et al. [10]	2011	Prospective cohort study	356	712		Increased hazard ratio in IBD group
Haapamaki et al. [49]	2011	Cross-sectional study	2831		Finnish National Health insurance registered IBD patients	Higher prevalence of CAD in IBD patients, especially in women
Rungoe et al. [50]	2013	Retrospective cohort study			4.6 million Danes aged ≥15 years	The risk of IHD was highest in the first year after IBD diagnosis
Sridhar et al. [44]	2011	Cross-sectional study			Hospitalized patients between the ages of 18-60 years included in the NIS 2006 database	Increased risk of mesenteric ischemia and venous thrombotic disorders with IBD

Cardiac ATED and IBD

Cardiovascular (CV) ATED is by virtue of IBD and its inclination toward thrombus formation. The thrombi may occlude the coronary arteries, especially the blood vessels already tapered due to atherosclerosis (CAD), resulting in MI and irreversible ischemic heart disease. This thrombophilia propensity of IBD can lead to poor cardiovascular outcomes and cardiac remodeling, which accounts for increased CV mortality and morbidity. 

A case-control study published in 2008, sourcing data from the University of Manitoba database by Bernstein et al., found that the incidence of cardiac ATED is higher in both males and females suffering from IBD [39]. Kristensen et al. conducted a nationwide Danish cohort study on around 20,795 patients with new-onset IBD and a mean age of 40.3 years who were matched with 199,978 controls according to age and sex and concluded the overall increased risk of CVD, including IHD, MI, and cardiovascular death in IBD patients, especially during active flares and persistent IBD [43] (Table 1). The same Danish team of authors also found the increased need for hospitalization for HF patients with IBD and found it to be strongly interdependent on the active flare-ups of the disease [46]. Another meta-analysis included all controlled observational studies, including data from Medline, Cochrane Library, Embase, and international conference abstracts that pertained to the risk of ATED and CV mortality in adult patients with IBD. This metanalysis highlighted no increased risk of CV mortality. Nonetheless, it associated IBD with an increased risk of IHD [47]. 

Studies have demonstrated that IBD is also commonly associated with ASCVD, a pathology often responsible for the higher CAD risk in IBD populations. For example, a historical cohort study conducted from 2004 to 2010 included 131 IBD patients (54 CrD and 77 UC subjects) diagnosed with CAD by cardiac catheterization, matched with 524 non-IBD controls with CAD. Patients with IBD were diagnosed at a younger age than non-IBD patients, were less likely to be engaged in smoking, and had low BMI. It was interesting that post-PCI outcomes in patients with IBD and CAD were similar to non-IBD controls [48]. A different group of authors sourced data from the Finnish National Health Insurance Register used 2831 IBD patients to complete generic 15D and disease-specific IBD questionnaires and reported a higher prevalence of CAD in IBD patients, especially in women [49]. A Danish population-based cohort study done from 1997 to 2009 by Rungoe et al. observed an increased risk of CAD (2.13 incidence rate ratio; 95%CI, 1.91 to 2.38) within the first year of IBD diagnosis [50].

In contrast to the studies above, other studies beg to differ. Surprisingly, these studies found controversial results that built a bone of contention for the researchers. For example, Sridhar et al. also analyzed and investigated other CVDs like CAD, CVA, and PVD but did not find any increased risk due to IBD in their cross-sectional study; instead they found a reversed relationship between these diseases and IBD [44]. Another study revealed a similar conclusion. A systematic review of studies between 1965 and 2006 was done by Dorn et al. that provided indirect evidence that IBD is not associated with increased CV mortality [51] (Table 2). Conversely, another meta-analysis that reviewed over 35 articles on PubMed and Embase published between 1941 and 2011 had a similar finding, in addition to the fact that the increased rates of death in IBD patients were found to be due to other causes such as colorectal cancer, pulmonary disease, and nonalcoholic liver disease, rather than in CVDs [52].

**Table 2:** Studies not supporting the association between inflammatory bowel disease and cardiovascular disease. IBD: inflammatory bowel disease; ATED: arterial thromboembolic diseases; CrD: Crohn’s disease; UC: ulcerative colitis; CV: cardiovascular; NIS: National Inpatient Database.

Author and reference	Year of publishing	Study design	Cases with IBD	Cases not with IBD	Population	Conclusion
Sridhar et al. [44]	2011	Cross-sectional study			Hospitalized patients between the ages of 18-60 years included in the NIS 2006 database	No increased risk of other cardiovascular diseases in IBD patients
Dorn et al. [51]	2007	Systematic review	4532 CrD and 9533 UC		Eleven studies with IBD were published between 1965 and 2006	IBD is not associated with increased CV mortality
Bewtra et al. [52]	2013	Systematic review			Thirty-five articles on PubMed and Embase were published between 1941 and 2011	Increased rates of death in IBD patients were found to be due to other causes
Fumery et al. [47]	2014	Meta-analysis			Thirty-three studies enrolling 207,814 IBD patients and 5,774,898 controls	No increased risk of ATED or CV mortality

Nevertheless, these studies chronicled CV mortality and not morbidity as the endpoint that indirectly depicts CVD risk. IBD affects younger people that are diagnosed and are under regular medical follow-up. Therefore, they are expected to be screened and managed for the CVD risk factors earlier than the average population, which might explain the controversial CV mortality outcomes. Early prevention is probably why developing typical CVD risk factors other than hypertension is rare. Thus, morbidity assessment like ischemic events during a lifetime like a stroke and MI can better depict the association rather than the CV death [53]. For example, Fumery et al. found no increased risk of arterial thromboembolism or CV mortality in IBD patients. However, they did find an increased risk for IHD and mesenteric ischemia [47].

There is a scope for future research in pediatric studies since the data pertaining to this topic of discussion is quite limited. Nonetheless, there are studies to provide an insight into pediatric IBD and its CVD risk. Nakano and his colleagues pursued a cross-sectional study to determine the link between total homocysteine and IBD in children, including 43 IBD subjects (27 CrD, 9 UC, and 7 IBD-U) and 46 controls. The study found that the total homocysteine levels were considerably higher in IBD patients than in controls, which indirectly provides evidence for the higher risk of development of CVD despite a young age [54]. A systematic review of population studies by Lazzarini et al. suggested an increased risk of ATED in children with IBD than in controls. The ATED events occurred during an active flare for almost all (82.8%) children of all age bars, and additionally, the findings were even more accentuated in children with UC [55].

In contrast to the above data, Dorfman et al. conducted a cross-sectional study on the late adolescent IBD population consisting of 2372 cases identified out of 1,14,4213 Israeli Jewish adolescents (mean age 17.1 years), comprising 1612 cases of Crohn's disease (68%). The study aimed to investigate the link between CVD and IBD further but did not find any significant risk factors for CVD among these subjects [56]. Another study examining pulse wave velocity (PWV) as an indicator for arterial stiffness was conducted by Lurz et al. on 25 children (10 UC and 15 CrD) with a mean disease duration of 2.8 years and found that none of them presented with the classic CVD risk factors and revealed that all children had PWV under the 95th percentile and the multivariate analysis did not show any link with inflammatory markers nor the disease duration or activity whatsoever [57] (Table 3). A retrospective review conducted at a tertiary care hospital analyzed around 532 children and young adults hospitalized with IBD with colonic involvement for the incidence of ATED. The detailed analysis revealed the increased risk of ATED and VTED, including pulmonary embolism and its complications, recurrence and sustenance of the events, and the need for long-term anticoagulation, which appears relatively safe in IBD with active colitis. Furthermore, inherited and acquired thrombophilias accentuate the ATED risk [58]. Therefore, all risk factors and tendencies must be stratified, and all high-risk individuals must receive timely prophylactic anticoagulation [59].

**Table 3:** Studies concerning the association between inflammatory bowel disease and cardiovascular disease in children. IBD: inflammatory bowel disease; ATED: arterial thromboembolic diseases; CrD: Crohn’s disease; CVD: cardiovascular disease; VTED: venous thromboembolic disease; UC: ulcerative colitis; PWV: pulse wave velocity.

Author and reference	Year of publishing	Study design	Cases with IBD	Cases without IBD	Population	Conclusion
Barclay et al. [45]	2010	Literature review	154		Pediatric newly diagnosed IBD patients	Increase in the relative proportion of strokes in CrD cases
Zitomersky et al. [58]	2013	Retrospective review	532		Children with IBD with colonic involvement	Increased risk of ATED and VTED, including pulmonary embolism
Nakano et al. [54]	2003	Cross-sectional study	43	46		Elevated plasma homocysteine is a consequence of IBD in children
Lazzarini et al. [55]	2011	Systematic review			Medline, LILACS, EMBASE, POPLINE, CINAHL	Increased ATED events during active flares
Dorfman et al. [56]	2020	Cross-sectional study	2372	1,14,4213	Jewish Israeli adolescents (mean age 17.1 years)	Significant risk factors for CVD were not present in adolescents with IBD
Lurz et al. [57]	2017	Prospective cohort study	25		Children (10 UC and 15 CrD) with a mean disease duration of 2.8 years	Children had PWV under the 95th percentile, and the multivariate analysis did not show any link with inflammatory markers

VTED and IBD

There is overwhelming evidence that IBD patients are at an increased risk of venous thromboembolism. Inflammatory markers promote thrombosis by creating a prothrombotic state, making veins more susceptible to thrombi formation. The concept has become widely accepted; therefore, early prophylaxis and treatment of VTED are an integral part of managing IBD.

A prospective cohort study published in 2014 with a cohort size of 1708 IBD patients, 648 CrD, and 1060 UC patients showed a VTED incidence of 1.03 per thousand patient-years after being followed for over 35 years, with a cumulative risk of 1.5% at the end of 15 years. This increased risk was higher in males but similar for UC and CrD. The VTED risk in UC patients was associated with the extensive location of the disease (OR = 3.25, 95%CI: 1.13-9.35), the fulminant episodes during the disease course (OR = 4.15, 95%CI: 1.28-13.5), smoking (OR = 3.46, 95%CI: 1.14-10.5), and the need for steroids (OR = 2.97, 95%CI: 0.99-8.92), but not with age at onset [60]. Another meta-analysis was conducted to include all controlled observational studies, including data from Medline, Cochrane Library, Embase, and international conference abstracts on the risk of VTED and ATED and CV mortality in adult patients with IBD, which highlighted the increased risk of VTED [47]. Furthermore, another cross-sectional study by Sridhar et al. strongly correlated IBD with venous thrombotic disease (1.38 OR adjusted; 95%CI, 1.25-1.53) [44].

Peripheral ATED and IBD

Peripheral arteries can be tapered and occluded owing to atherosclerosis and the prothrombotic state of IBD, like occlusion of mesenteric arteries can lead to mesenteric ischemia. A meta-analysis conducted by Singh and his colleagues analyzed nine studies, highlighted the increased risk of VTED, and found an increased risk of mesenteric ischemia [42]. Supporting the evidence further, a retrospective cohort study with 17,487 IBD patients and 69,948 controls observed a similar risk increase for acute mesenteric ischemia in both UC and CrD patients with a female predominance [40]. The cross-sectional study by Sridhar et al. also strongly correlated IBD with acute mesenteric ischemia (3.4 OR adjusted; 95%CI, 2.9-4.0) [44]. Contrastingly, the same systematic review by Singh et al. did not find any correlation between IBD and the risk of other peripheral vascular diseases (PVD) [42].

The CVD Paradox

Although several studies have been ushered and channeled into elaborating the risk of CVD in patients with existing IBD, the results and outcomes of the research studies have been irreconcilable with many undiscovered and unexplained factors [47]. It is a well-established fact that the higher the risk of classic cardiovascular risk factors, the higher the risk of CVD mortality and morbidity [29]. However, this comprehension holds untrue when it comes to IBD patients. Various studies prove that classic cardiovascular risk factors are lower in IBD patients than in the general population [10,61-63]. For example, a higher body mass index (BMI) is a significant risk factor for CVD, but body weight and BMI are surprisingly lower in IBD patients [62,64]. The lipid profile and lipoprotein composition are altered in IBD in the opposite direction of what is expected to be found in classic CVD patients [62]. People with IBD also have a lower incidence of obesity, hypertension, and diabetes [10]. Nevertheless, there is an increased risk of CVD witnessed in IBD patients, notwithstanding the lack of traditional risk factors, indicating that these factors do not impact the development of CAD in IBD subjects [10].

Therapeutic and prognostic implications

Pharmacological Treatment 

The standard treatment for IBD include 5-aminosalicylic acid (5-ASA), corticosteroids (CS), immunomodulators like thiopurines (TPs), methotrexate (MTX), calcineurin inhibitors, and Janus Kinase (JAK) inhibitors, and biological agents that include pro-inflammatory cytokine (TNF-α and IL-12/23) inhibitors and integrin antagonists [64].

Amino salicylates include sulfasalazine, a pro-drug of this class, with 5-ASA (active component) and sulphapyridine (carrier). 5-ASA works by hindering the functioning of white cells and cytokines by intruding on their metabolism and their ability to scavenge oxygen. These drugs have been clinically used to treat UC for decades. The following data highlights its efficacy in modifying CVD risk [64]. Rungoe et al. performed a Danish population-based study to examine 28,833 IBD patients from 1997 to 2009 and found that the subjects that received 5 ASA treatment had a decreased risk of IHD in contrast to those who never received 5-ASA [65].

Conversely, Zanoli et al. conducted a small-sized longitudinal study on 32 IBD subjects; 14 were treated with only 5-ASA, 11 were treated with CS and azathioprine, and seven were treated with TNF-α, as opposed to 30 matched controls. The study's endpoint was measured by PWV 3.4 ± 0.5 years later. This study demonstrated a significant increase in carotid-femoral PWV after follow-up in IBD subjects treated with 5-ASA, indicating arterial stiffness [66].

Oral CS has been known to induce remission in the case of active flares. These drugs have been clinically used for IBD since the 1950s. CS has intranuclear receptors and interacts with pro-inflammatory transcription factors to modulate the effects of the inflammatory system. Additionally, it promotes the expression of the anti-inflammatory genes in the nucleus, further emphasizing its anti-inflammatory effect [64]. These drugs are efficacious in mild to moderate IBD with extensive lesions [67]. The following study highlights its impact on CVD risk. The same Danish study mentioned above by Rungoe et al. also found a similar yet more substantial cardioprotective effect in patients treated with oral corticosteroids (CS) [65]. Howbeit, contrasting to the above study, another article describes the increased risk of CVD with prolonged CS treatment [8].

TPs belong to the class of immunomodulators that alters the functioning of inflammatory cells. TPs such as azathioprine interfere with T-lymphocyte proliferation and synthesize deoxyribonucleic acid (DNA). It is known to have a favorable therapeutic effect that reduces the need for hospitalizations and surgeries in IBD patients. TNF-α is one of the most critical cytokines that indulge in inflammation initiation and propagation. Anti-TNF-α therapies like Adalimumab and Infliximab inhibit TNF-induced inflammation and tissue damage in patients intolerant or do not respond well to CS and immunomodulators and are steroid-dependent [64]. A lower IHD incidence was also observed upon treatment with thiopurines or TNF-α blockers [65].

Conversely, Zanoli et al. did not find any increased carotid-femoral PWV with CS and azathioprine nor with anti-TNF-α agents [66]. Although TNF-α antagonists are extensively used in IBD management as they are known to reduce the inflammatory burden and aid mucosal healing in some; however, data indicating its potential cardioprotective effect on decreasing the risk of CVD in IBD patients is scarce. A prospective cohort study to support this argument comes from the Danish population. Anderson and his colleagues addressed this risk of CVD in terms of IHD and CVA and followed the IBD patients for 11 years post the initiation of their anti-TNF therapy from 1999 to 2010. The study yielded an adjusted HR of 0.85 (95%CI: 0.59-1.24) for IHD, whereas the risk of CVA associated with TNF-α antagonists was 1.42 (95%CI: 0.82-2.45). This study advocates a protective effect of TNF-α antagonists on IHD. However, in contrast, the use of TNF-α antagonists might be a risk factor for CVA, although none of the values (to support the latter statement) were statistically significant [68].

Another large retrospective cohort study with 1986 statin-exposed and 9871 unexposed subjects implied to investigate the role of statins in reducing inflammation and surprisingly revealed an 18% reduction in the need to initiate oral CS treatment in IBD patients (HR = 0.82; 95%CI: 0.71-0.94), and an even more significant decline in patients with UC (HR = 0.75; 95%CI: 0.62-0.91) [69].

Other biologics such as vedolizumab and ustekinumab have not reported any adverse events according to the systemic review conducted by Solitano and his colleagues. Also, ATED events rarely occur in patients with UC after therapy with tofacitinib [70]. Another meta-analysis conducted by Zhou et al. suggests that vedolizumab and infliximab are the most effective amongst the biologics, with tofacitinib and fecal microbiota transplantation as alternatives [71].

Although the studies mentioned above add knowledge and perspective to the current literature existing on the catastrophic damage IBD has on CV outcomes and disease severity; howsoever, the need for more statistically significant studies to determine the steroid use and risk, anti-TNF exposure, and risk of CVA, IHD, and MI to emphasize the issue further and its management is palpable [4,51,68].

Dietary Treatment

Dietary intake in IBD is significant yet controversial based on the existing literature. The dietary protocols that the patients are advised to follow judiciously aim to limit their dietary fiber intake, like fruits and vegetables, which in fact increase their carbohydrate intake, inciting microbiota dysbiosis and damage [72]. A diet rich in fiber and limited in carbohydrates may decrease inflammation and support IBD remission [73]. It is not wrong to say that reduced inflammation equals reduced CVD risk, which is a bonus [74].

Additionally, the Mediterranean diet (MD) explained by Szuber et al. in a recent narrative review published in August 2021 was advantageous for IBD patients no matter how intolerant, as it benefits by decreasing CVD risk. This study also points out the incompetence of the western diet, which in fact, adds to the increased risk of IBD and CVD. Thus, IBD patients should avoid the western diet to their ability [65].

Furthermore, this review also highlights the controversial data on the role of vitamin D and calcium in CVD development and the further need to explore this aspect of IBD management [65]. Most studies showed the preventive activity of calcium in developing CVD [75] in contrast to others [76]. Moreover, calcium is known to positively impact the blood pressure and lipid profile, favoring a decreased risk of atherogenesis [77].

From all the data, the takeaway point, as mentioned earlier, is that the first and foremost goal of treatments should be to reduce inflammation as early as possible to prolong remissions and prevent active flares. This fact should be emphasized as much as possible, for it serves as the foundation for decreasing the CVD risk in IBD patients.

Therefore, it is imperative to work up the case with careful evaluation and investigations once the patient has been diagnosed with IBD to know the extent, severity, and location of the inflammatory lesions and deliberate the existence of potential complications with regular follow-ups and management. A thorough evaluation will guide the choice of the best course of action and help clarify therapeutic and prognostic implications. There should be routine counseling for high-risk populations (e.g., those with active disease, women, and young patients) about modifying aggressive risk factors and adhering to therapeutic guidelines [65].

Limitations

Although there are many potential mechanisms explained in this review that interlink CVD and IBD but there can be other confounding variables that could have been involved in the development of poor cardiovascular outcomes and could explain the co-occurrence of CVD with IBD, given the multitude of etiologies that involve more than one causative factor. The review article is limited to the effective mechanisms and treatments to link IBD with CV mortality and morbidity. However, it does not provide detailed insight into the minor mechanisms or management protocols. This association should be investigated further with additional research and in-depth analysis.

## Conclusions

Over the years, many studies have been conducted and reviewed extensively to know the CVD risk in chronic inflammatory conditions like IBD. This review has incorporated data extracted from several articles and comprehensively discussed the correlation between IBD and CVD and its clinical and therapeutic implications. IBD may commence, catenate, and congregate atherosclerosis leading to ASCVD. An overwhelming majority of recent studies have shown the increased risk of CVD in IBD patients due to the multitude of etiologies and pathways led by ongoing chronic inflammation being the primary culprit in hindsight. Interestingly, despite the lack of traditional risk factors for CVD, IBD paradoxically has significantly been linked to increased risk of IHD, CVA, and MI, especially during active flares of the disease, with an ambiguous risk for cardiovascular mortality, which implies the need to keep IBD in remission for as long as possible with appropriate, timely treatment. This increased risk of poor cardiovascular outcomes can be attributed to the altered coagulation profile leading to ATEDs requiring prophylaxis. Specific high-risk populations like females, active disease individuals, and pediatric age groups have shown a comparatively higher risk of CVD making it crucial to start treatment and potential prevention at an early age. Recent data have also demonstrated that microbiota alterations (dysbiosis) negatively impact atherogenesis, leaving significant scope for exploring dietary foods that maintain homeostasis in the microbiota as a potential prevention and treatment. A favorable impact has been seen with salicylates. However, a debatable impact is seen with steroids, azathioprine, and anti-TNF-α agents, which remarkably effectively take off the inflammatory burden but lead to conflicting CV outcomes that need further attention. Also, there is insufficient data to explore the link between pediatric IBD and CVD, leaving room to elucidate this regard in-depth. Different avenues of research can discuss and explore this link to offer potential preventive and treatment strategies to decrease CV morbidity and mortality in IBD patients.
